# Supplementary material for: Determinants of abortion among youth 15–24 in Ethiopia: A multilevel analysis based on EDHS 2016
Source: PLoS One. 2021 Mar 12;16(3):e0248228. doi: 10.1371/journal.pone.0248228 (PMC7954319; doi:10.1371/journal.pone.0248228)
Supplement: Attachment — Submitted filename: Response to Reviewers.docx [file pone.0248228.s002.docx]

Firstly, we are grateful to esteemed reviewers, board of editors and all involved stakeholders in making this work eligible for publication in PLOS ONE journal.

In this section, we tried respond to each and every concerns raised by the esteemed reviewers. We provided the response in the similar sequence they appeared in the feedback form. Some of the points might be raised differently by reviewers and the response also considered them accordingly.

1. **Reviewer #1**

It is suggested to change the title to ‘determinants of pregnancy termination among youth 15-24 in Ethiopia’ because the paper did not talk about pregnancy termination except the prevalence of it

***Determinants of abortion among youth 15-24 in Ethiopia:* A multilevel analysis based on EDHS 2016**
⎫ The introduction lacks sound justification to undertake the study, for example, the severity of the problem, impacts of the problem if not studied

Before 2005, the Ethiopian government legalized only the procedure of abortion, in case it is required to save the life of a woman(16). During that time, abortion-related complications were 32% of all maternal deaths(17). The current law allows abortion in case of rape, incest, and fetal impairment; if a woman has physical or mental disabilities; or if she is younger than 18 years old. It is the policy that semi liberalizes abortion(17)(18). After this, the number of abortions increased by some amount especially among the age group of 15-19. From other pieces of evidence, one in ten youth reported abortion concerning different socio-demographic factors(19). In 2014, the number of adolescents seeking post-abortion care due to clandestine abortion was greater than those aborted legally (50% vs 18%); between learned and unlearned (14% vs 5%). Data in the same year showed, all types of abortion of all ages increased from 27% to 53%, and was 20% for adolescents. And we observed 96,000 induced abortions and 35,000 clandestine abortions(20). However, the fact that most Ethiopians are religious and have culturally strong identities, we might saw clandestine abortion with complications, and the real could be very large(4). Thus, considering these backgrounds, more work remained ahead and studies like this might be helpful to increase information related to these gaps for future policymaking decisions so that adequate youths might obtain the service. Therefore, determining abortion among youths and its associated variables expected to be a great asset for this state of affairs in Ethiopia
⎫ The discussion part did not include the limitation of the study, the study has serious limitations, for example, disproportionate sample size among regions, unjustified age, and socioeconomic status category, which statically changed the results of the study

EDHS follow a kind of data collection and methodologies which are internationally standard and scientifically applied in every country so that most limitation might be tolerated. The clustering effect might be another issue during sampling. Furthermore, the economic status classification was dependent on the country’s economic status. However, to account for the limitations, we applied weighting techniques, and Segregated or multilevel analysis to handle variations among clusters
⎫ There is a serious grammar problem majorly in the methodology and result part

# Methods and materials

## Design

A cross-sectional study design deployed in Ethiopia based on the data from Ethiopian demographic health surveillance (EDHS) 2016.

## Setting and participants

World Bank and the national bank of Ethiopia estimated the total population of Ethiopia at 112.0 million people in 2019. The country is divided into 9 regions (Tigray, Afar, Amhara, Oromia, Somali, Benishangul, SNNPR(south nation nationalities people’s region), Gambela, and Harari) and 2 city administrations (Addis Ababa and Dire Dawa). The administration levels went from regions, zones, and through woredas

(21). We downloaded EDHS 2016 dataset for this study purpose and extracted youth females of age 15-24 considering only those who had complete records and then cleaned and made the data ready for the analysis.  In this process, we extracted 6,401 samples  (22). EDHS collects data on fertility and childhood mortality levels, fertility preferences, awareness, approval, use of family planning methods, maternal and child health, domestic violence, knowledge, and attitudes toward HIV/AIDS and other sexually among the adult population. The frame of the Population and Housing Census (PHC) contains a complete list including information about the enumeration area (EA) location, type of residence (urban or rural) and the estimated number of residential households which developed for this purpose by the Central Statistical Agency (CSA) used(22). We accessed the data from the Demographic and Health Survey (DHS) website (http://www.dhsprogram.com) which request registration for permission. The data we got then used only for the research purpose. We kept all data confidential, and no effort was made to identify households or individuals.  EDHS approved by the Ethiopian Health Nutrition and Research Institute (EHNRI) Review Board and the National Research Ethics Review Committee (NRERC) at the Ministry of Science and Technology, Ethiopia. As published in the survey report of 2016, they collected verbal informed consent from participants, and the purpose of the study was clear for participants (22). Participation in the survey was voluntary and they respected the right to decline.

## Study variables and data management

The outcome variable for this study was ‘abortion’ extracted from an EDHS question assessed through ‘have you ever had a termination of pregnancy’ with the responses ‘yes’ if the women ever had an abortion and otherwise ‘no’ as a binary outcome.   Abortion is usually mean any pregnancy outcome of a miscarriage, abortion, or stillbirth;(23) (24) (25). Considering all the limitations of the secondary data as they are, for this study purpose, the term abortion was appropriate as an all-inclusive indicator. The exploratory variables are individual or group variables showing both mother and child, every socio-demographic variable used in the selected EDHS dataset. After downloading the dataset and including it in the study according to the criteria, we cleaned data in Stata v. 15.0.  The data then weighted as per sampling weight, primary sampling unit, and strata before analyzing in Stata 15.0

Finally, we examined abortion in 2016 datasets and discovered the correlation of independent with outcome variables. Individual and group-level predictors of abortion examined using multilevel logistic regression on pooled data from the datasets.  Significance level maintained at p<0.05 with 95% confidence intervals (CI). Before the multilevel logistic regression application, we checked all assumptions. Each variable checked on bivariate before introducing into the consecutive multilevel logistic regression models where 0.2 was used to include variables to models.

# Results

We pooled 6,401 youth female population from EDHS 2016 dataset picked for the current study to examine abortion status in age 15-24. The proportion of abortions became 2.5%. Community-level weighted frequency analyses showed the larger number of participants were from Oromo (36.3%), Amhara (22.5%), and SNNP (20.37%) regions. Three-fourth of the participants were from urban residences.  At the individual level, the age group of 15-19 was accounted for 55% of all participants.  More than half of the participants were only learned primary education (54.25%) and no education also accounted for 20%. More than half (56%) of youths included in this study were single and 39% were married. Eighty-four percent of youths started sexual intercourse earlier than 18years and 72% of them had no work during the survey; however, 48% of them were in the rich wealth status category and 32% in poor. The birth was not experienced in70% of youths and half of them had no media exposure

## Multilevel analysis

The null model had 15.7% of the total abortion variations coming from the variability among clusters and the rest from individual differences. The clustering effect shown up here more directed us to take multilevel analysis.   The median odds ratio of abortion in the null model was 2.1 affirming the differences among the clusters. This means we cannot be sure if the results of two randomly picked clusters different or similar as we cannot rule out the between cluster variations.

We followed four model building steps to account for the inter-cluster variation stated above. The null model is an intercept only model but, has given the clue of continuing model development. In model I which is the individual level, females in the age group 20-24 were 2.5 times more likely to carry out abortion with an AOR of  2.5 (1.6-3.8). Considering birth in the last five years, female with at least one birth showed 35% reduced abortion with an AOR of  0.65 (0.44-0.96), and those who gave to 2-5 births had  69% reduced abortion with an AOR of 0.31 (0.18-0.55) compared to no birth category. Similarly, the odds of being in the abortion group was 45% reduced for age ≥18 years with an AOR of 0.55 (0.36-0.82) than those <18. In contrast to this, youths who married had 32 times more likely to carry out abortion with an AOR of 32(14-70), and those who made divorce were 16 times more likely to experience abortion with an AOR of 16.6(6-44) compared to single.

And in model 2 which is a community level, Amhara, Benishangul, SNNP, and the Gambella had 66%, 71%, & 54% reduced abortion with AOR of 0.34(0.15-0.75), 0.29 (0.11-0.75), 0.46(0.23-0.94), and 0.28 (0.10-77) respectively compared to the Somali region.

In the ultimate or mixed model, females in the age group 20-24 were 2.5 times more likely to carry out abortion with an AOR of  2.5(1.6-3.8) relative to age 15-19. The odds of abortion among females with good wealth status were 1.7 times more than the odds of poor wealth status females with an AOR of 1.7(1.0.4-2.8). Considering birth in the last five years, females with at least one birth had 35% reduced abortion with an AOR of  0.65(0.44-0.96) and those who gave 2-5 births had  69% reduced abortion with an AOR of 0.31(0.18-0.55) compared to no birth category.

The odds of being in the abortion group were 50% reduced for age ≥18 years old with an AOR of 0.50(0.33-0.76) compared to age <18. And as in model 1, youths who married had 38 times more likely to report abortion with an AOR of 38(17-84) and those who divorced were 20 times more likely to have an abortion with an AOR of 20(7-55) compared to single marital status. And for community level, youth females in the Amhara region were 69% reduced abortion with an AOR of 0.31(0.11-0.85) and those living in Benishangul had 70% reduced abortion with an AOR of 0.30(0.12-0.77)

2. **Reviewer #2**

(1) Disaggregating the pregnancy termination variable to focus specifically on pregnancy termination (i.e. induced abortion). This will probably require analysing the data again.

***Determinants of abortion among youth 15-24 in Ethiopia:* A multilevel analysis based on EDHS 2016**

The outcome variable for this study was ‘abortion’ extracted from an EDHS question assessed through ‘have you ever had a termination of pregnancy’ with the responses ‘yes’ if the women ever had an abortion and otherwise ‘no’ as a binary outcome.   Abortion is usually mean any pregnancy outcome of a miscarriage, abortion, or stillbirth;(23) (24) (25). Considering all the limitations of the secondary data as they are, for this study purpose, the term abortion was appropriate as an all-inclusive indicator. The exploratory variables are individual or group variables showing both mother and child, every socio-demographic variable used in the selected EDHS dataset. After downloading the dataset and including it in the study according to the criteria, we cleaned data in Stata v. 15.0.  The data then weighted as per sampling weight, primary sampling unit, and strata before analyzing in Stata 15.0

(2) Situating the analyses within the literature in a more nuanced and coherent way- on pregnancy termination (e.g., the authors cite in the introduction, a paper by Ganatra et al, that reports data on abortion according to a spectrum of safety, but the authors report this within a binary- which, once again, raises questions about how the analyses are presented later on), on pregnancy termination in Ethiopia (for example, see literature published in PLOS One: https://doi.org/10.1371/journal.pone.0235382) and Sub-Saharan Africa ( https://doi.org/10.1371/journal.pone.0235329), adolescents/young people and pregnancy termination in Ethiopia (https://www.guttmacher.org/news-release/2018/first-study-incidence-abortion-among-ethiopian-adolescents-released, https://www.guttmacher.org/fact-sheet/adolescent-abortion-ethiopia, https://doi.org/10.1186/s12889-019-6845-7, <https://doi.org/10.1016/j.jadohealth.2017.12.015>).

Abortion is an expulsion of pregnancy tissue and products of conception either intentional by acts or spontaneous(1). Worldwide abortion is the proven cause of maternal death(2). Abortion procedures can be safe or unsafe but studies associated every type of it with the risk of youth mother and fetus deaths. Previous shreds of evidence showed that pregnancies from youth subjected to frequent termination because of physically and psychologically incomplete growth and development(3). The problem resulting from abortion increase as access has been also influenced by laws and cultural aspects of the communities across the world(4). Legalizing abortion showed improvement in access and usage but do not reduce the number of estimated abortions(5).

Abortion has different outcomes on the future of youth mothers and the life of the fetus.  Other than termination, conceiving during the youth age by itself is a higher risk and correlated with adverse pregnancy outcomes (6). Studies highlighted that youth pregnancies are additionally associated with hypertension, eclampsia, premature onset of labor, fetal deaths, premature, and pre-eclamptic toxemia delivery outcomes(7).

Individual and community-level factors are responsible for engagement in abortion and pregnancy terminations among which socio-demographic status, resources, early marriage, and sexual intercourse dominated(8)

. Family background, age, social environment, emotional wellbeing, educational status, employment status, and relationships were the variables significantly associated with early youth pregnancy and bbortionearly youth pregnancy antatus, resources, earily marriage and sexual intercourse dominated ntry. abortion(9) (10), (11), (12). A study in Gahanna showed education, religious beliefs, health, economic factors, and family made a maximum impact where other factors determined the decision to undergo abortion (13). Another study indicated good knowledge of abortion rights and services increase usage, even so, quality, accessibility, and safeties of the abortion were also assumed to affect the decision to pick the service(14). In Sub-Saharan countries, women who can make reproductive health decisions are more likely to undergo abortion services(15).

Before 2005, the Ethiopian government legalized only the procedure of abortion, in case it is required to save the life of a woman(16). During that time, abortion-related complications were 32% of all maternal deaths(17). The current law allows abortion in case of rape, incest, and fetal impairment; if a woman has physical or mental disabilities; or if she is younger than 18 years old. It is the policy that semi liberalizes abortion(17)(18). After this, the number of abortions increased by some amount especially among the age group of 15-19. From other pieces of evidence, one in ten youth reported abortion concerning different socio-demographic factors(19). In 2014, the number of adolescents seeking post-abortion care due to clandestine abortion was greater than those aborted legally (50% vs 18%); between learned and unlearned (14% vs 5%). Data in the same year showed, all types of abortion of all ages increased from 27% to 53%, and was 20% for adolescents. And we observed 96,000 induced abortions and 35,000 clandestine abortions(20). However, the fact that most Ethiopians are religious and have culturally strong identities, we might saw clandestine abortion with complications, and the real could be very large(4). Thus, considering these backgrounds, more work remained ahead and studies like this might be helpful to increase information related to these gaps for future policymaking decisions so that adequate youths might obtain the service. Therefore, determining abortion among youths and its associated variables expected to be a great asset for this state of affairs in Ethiopia

(3) We also need to understand the study context better: what is the law in Ethiopia on pregnancy termination, how does it treat young peoples' access to abortion? What are the social barriers?

Before 2005, the Ethiopian government legalized only the procedure of abortion, in case it is required to save the life of a woman(16). During that time, abortion-related complications were 32% of all maternal deaths(17). The current law allows abortion in case of rape, incest, and fetal impairment; if a woman has physical or mental disabilities; or if she is younger than 18 years old. It is the policy that semi liberalizes abortion(17)(18). After this, the number of abortions increased by some amount especially among the age group of 15-19. From other pieces of evidence, one in ten youth reported abortion concerning different socio-demographic factors(19). In 2014, the number of adolescents seeking post-abortion care due to clandestine abortion was greater than those aborted legally (50% vs 18%); between learned and unlearned (14% vs 5%). Data in the same year showed, all types of abortion of all ages increased from 27% to 53%, and was 20% for adolescents. And we observed 96,000 induced abortions and 35,000 clandestine abortions(20). However, the fact that most Ethiopians are religious and have culturally strong identities, we might saw clandestine abortion with complications, and the real could be very large(4). Thus, considering these backgrounds, more work remained ahead and studies like this might be helpful to increase information related to these gaps for future policymaking decisions so that adequate youths might obtain the service.

(4) Language: there is a lot of 'youth' or 'teen' used in this article- even where it is not applicable or appropriate (e.g., ages 15-24. what is understood by 'teen' here?). Youth as a 'catch all' term obfuscates- especially if the data are specific to young women and girls' experiences? If so, that needs to be accounted for clearly in the text- and the gendered implications of this

We mistakenly used the term ‘**teen’** and ‘**youth**’ interchangeably in some of the contexts. After, it brought to our attention by esteemed reviewer #2; we analyzed and agreed on the term ‘**youth’** instead and changed all of it in the area where it was not appropriate.

Thank you once again for all the critical comments and your time. We appreciate your expertise work and professionalism of both reviewers and really grateful for that.

Authors,
